# Enumeration and Genomic Confirmation of Viable Shiga-Toxin-Producing *Escherichia coli* from Ground Beef by Integrating Serial Plating with Long-Read Sequencing

**DOI:** 10.3390/pathogens15060573

**Published:** 2026-05-27

**Authors:** Katrina L. Counihan, Shannon Tilman

**Affiliations:** Eastern Regional Research Center, United States Department of Agriculture, Agricultural Research Service, Wyndmoor, PA 19038, USA

**Keywords:** *Escherichia coli* O157:H7, food safety, serial plating, long-read sequencing, enumeration, bioinformatics, identification, single colony

## Abstract

Consumption of food contaminated with Shiga toxin-producing *Escherichia coli* (STEC) causes thousands of illnesses in the United States annually. Long-read sequencing could reduce the time needed to test food for STEC, but sequencing is not quantitative and cannot differentiate between live and dead bacteria. Therefore, this study investigated combining serial plating with long-read sequencing to quantify only live STEC in a sample. Ground beef was inoculated with STEC and homogenized with a stomacher. The liquid was filtered to remove eukaryotic cells and then centrifuged to pellet the bacteria. Serial dilutions of the pellet were plated on selective agar, and single colonies were subsequently sequenced. Initial experiments revealed that processing samples at room temperature led to a 1 log increase in STEC levels from the initial inoculated concentration, confounding accurate enumeration. In subsequent experiments, samples and reagents were kept cold, and the amount of STEC recovered did not significantly differ from the amount inoculated. The DNA extracted from a single colony provided enough data to identify all virulence genes of interest multiple times. The amount of STEC in a sample could be quantified down to 1 cfu g^−1^. Quantification of STEC in food samples using this method would improve risk assessment and guide mitigation efforts in industry.

## 1. Introduction

An estimated 48 million people become ill annually in the United States (U.S.) due to foodborne pathogens [1]. Shiga toxin-producing *Escherichia coli* (STEC) is a foodborne bacterium that typically produces diarrhea and vomiting in infected individuals but may also cause severe pathologies such as hemorrhagic colitis or hemolytic uremic syndrome [2]. The incidence rate of STEC infections has been increasing since 2018 and is 6.6 per 100,000 people, with 20.4% of cases resulting in hospitalization and 0.4% being fatal [3]. Children under 5 are more likely to become ill and had an incidence rate of 17.8 per 100,000 people in 2022 [4]. Furthermore, the impact extends beyond the human health cost, as significant economic losses can result from pathogen contamination of food. In the U.S. during 2021, over 15 million pounds of meat were recalled, and STEC contamination was responsible for two of those recalls [5]. The estimated annual cost of foodborne pathogen contamination to the U.S. economy is approximately $17 billion [6].

Humans acquire STEC through contact with feces-contaminated environments, animals, or people [7]. Foodborne infections are most commonly associated with the consumption of STEC-contaminated meat and leafy greens [8]. Ingestion of unpasteurized raw milk or its products, such as cheese, is increasingly becoming a source of STEC infections, as well [9]. Animals, including cattle, are natural reservoirs for STEC and can shed the bacteria into the environment in their feces, contaminating soil and water [8,10]. Contaminated soil, water, or manure used as fertilizer can contaminate produce [10]. STEC present in the gastrointestinal tract or on the skin of cattle or swine can be transferred to the carcass during skinning and evisceration [7]. Both meat and produce can also be contaminated by processing equipment [8]. When raw beef becomes contaminated by STEC, it is considered “adulterated” by the U.S. Department of Agriculture Food Safety and Inspection Service (USDA FSIS) [11].

Testing meat for STEC adulteration is performed by the USDA FSIS, and isolation and identification are accomplished through a combination of culturing, molecular methods, O-antigen typing, and matrix-assisted laser desorption/ionization time-of-flight (MALDI-TOF) mass spectrometry [12]. A food sample is considered adulterated if it tests positive for the *stx*, *eae*, *espK*, and *espV* genes and one of seven targeted O-antigens associated with STEC infections in humans [12]. Intimin, the product of the *eae* gene, mediates attachment to intestinal epithelial cells in the host [2]. The interaction of intimin with other virulence factors results in the formation of attaching and effacing lesions, within which cytoskeletal rearrangement and microvilli destruction occurs, causing diarrheal symptoms [2,13]. Shiga toxin 1 and 2, products of the *stx1* and *stx2* genes, inhibit protein synthesis and stimulate cytokine production, both causing cytotoxicity in the host [2,14]. The *espK* and *espV* genes are secreted effectors of the Type III Secretion System that have been associated with severe disease in humans and are primarily found in enterohemorrhagic *E. coli* (EHEC), which are strains of STEC [15]. *espK* facilitates persistence in the intestine, and *espV* is an effector from the avirulence A family whose function in disease is unknown [15]. In 2024, the USDA FSIS updated its STEC reference method to include testing for *espK* and *espV*, as including these genes reduces the number of presumptive positives that require further screening [15,16]. The STEC serotype most frequently associated with foodborne outbreaks is O157:H7 [17]. The *E. coli* serotype is determined by the O-antigen, which is located on the outer membrane, and the H-antigen, which is present on the flagella [18]. The USDA FSIS identification method takes at least four days to complete [12]. The time required for foodborne pathogen identification could be reduced by integrating long-read, whole-genome sequencing with culturing.

Oxford Nanopore Technologies’ long-read sequencers could be beneficial for foodborne pathogen testing because pathogen detection can be completed in hours instead of days through real-time analysis [19]. The sequencers are small and portable, allowing whole-genome analysis to be performed outside of traditional laboratories, and sequencing costs are generally lower than second-generation sequencing. Additionally, the accuracy of raw reads from nanopore sequencing has improved to >99.9%, which is comparable to short-read sequencing [20]. Previous work in our lab investigated the practicality of long-read sequencing for identification and characterization of *E. coli* O157:H7 in ground beef [21]. Inoculated ground beef was homogenized with media in a stomacher. The liquid was removed for DNA extraction and sequencing. The presence of the *eae*, *stx1*, *stx2*, *fliC*, *wzx*, *wzy*, and *rrsC* genes in sequencing data indicated *E. coli* O157:H7 contamination. The *fliC* gene codes for the flagellar H-antigen, and *wzx* and *wzy*, encoding a translocase and polymerase, respectively, are involved in O-antigen production [22,23]. The ribosomal 16S rRNA gene, *rrsC*, identifies the presence of any *E. coli* to the species level. However, despite high inoculation levels (10^7^ cfu g^−1^), all of the virulence genes needed to serotype *E. coli* could not be reliably identified in sequencing data from unenriched ground beef samples. The large quantity of bovine DNA outcompeted *E. coli* DNA for sequencing pores. Detection of very small concentrations of pathogen DNA within a high concentration of host DNA is a challenge that needs to be overcome.

An additional challenge for sequencing-based detection methods is the inability to differentiate between live and dead bacteria. Sequencing detects DNA from both live and dead organisms, but only live pathogens can infect and cause disease. Extracellular DNA and DNA in dead cells can persist for days to weeks [24,25]. Detecting DNA from dead bacteria could overestimate pathogen abundance [26] and potentially result in a costly and unnecessary food recall [27]. Therefore, culture-based methods remain the gold standard because bacterial growth indicates viability [28]. This issue must be resolved before sequencing can become a practical testing method.

Another disadvantage of both sequencing- and culture-based methods is that quantification of pathogens may be difficult or even impossible. Culture-based methods typically require an enrichment step to increase the pathogen concentration above the detection limit of the testing method being used, which prevents quantification of the initial pathogen concentration in the sample. Therefore, developing methods that bypass enrichment while retaining the viability selection of culturing is a key priority for advancing food safety testing. The most probable number procedure can estimate the number of bacteria in a sample, but it does not provide absolute quantification [29]. Sequencing-based quantification has been achieved through the addition of internal DNA standards of known concentration to samples. A correction factor can then be established to calculate the quantity of target DNA in a sample [30]. The amount of sequencing data produced has also been shown to correlate with DNA input, and further investigation may lead to additional quantification methods that do not rely on standards [31]. However, while these sequencing-based quantification methods show promise, they still cannot differentiate between live and dead bacteria, a fundamental advantage of our workflow, which maintains a culturing step.

The goal of this study was to determine whether serial dilution of *E. coli* O157:H7 contaminated ground beef onto selective media, followed by sequencing of single colonies, would result in both enumeration and identification. The objectives included evaluating whether processing temperature influenced the accuracy of bacterial recovery and determining if virulence genes of interest could be reliably detected in sequencing data from one colony.

## 2. Materials and Methods

### 2.1. Bacterial Culturing

*Escherichia coli* O157:H7 (ATCC strain 43895) was grown overnight in 10 mL tryptic soy broth (Oxoid Limited, Hampshire, UK) modified with novobiocin (mTSB; RPI Corporation, Mount Prospect, IL, USA) in an incubator at 42 °C ± 1 °C shaking at 180 rpm, which adheres to FSIS methods [12]. Optical density at a wavelength of 600 nm (OD 600) was measured with a Denovix DS-11 FX+ spectrophotometer (DeNovix Inc., Wilmington, DE, USA) to estimate bacteria concentration.

### 2.2. Small-Scale Experiment

A 2% sorbitol-mTSB (*w*/*v*) solution was prepared by adding D-sorbitol (Sigma-Aldrich, Saint Louis, MO, USA) to mTSB. Seven treatments were tested in three independent experiments. All treatments consisted of 3 mL cold 2% sorbitol-mTSB solution in sterile 7 oz Whirl-Pak^®^ filter bags (Austin, TX, USA). Three treatments were controls and included: media only, no meat inoculated with 10^3^ cfu *E. coli*, and 1 g of uninoculated meat. The remaining four treatments were prepared with 1 g of ground beef inoculated with *E. coli* to achieve final concentrations of 10^3^, 10^2^, 10^1^, or 10^0^ cfu g^−1^, respectively. All bags were homogenized with a Bag Mixer stomacher (Spiral Biotech Inc., Norwood, MA, USA) for 120 s on normal speed. Samples were sequentially vacuum filtered through a 60 µm steri-flip filter (EMD Millipore Corporation, Burlington, MA, USA) and then a 20 µm steri-flip filter (EMD Millipore Corporation). Each filter was washed with 1 mL of mTSB after the sample had passed through. The tubes with the resulting filtrate were then refrigerated at 4 °C for 1 h. If present, residual fat was removed from the surface of the filtrate with a sterile spatula. Tubes were then centrifuged at 130× *g* for 10 min and any residual fat was removed from the surface of the filtrate. The supernatant was transferred to a clean tube and centrifuged at 13,000× *g* for 5 min. The supernatant was discarded, the pellet washed with 1 mL of phosphate-buffered saline, (PBS; Boston BioProducts, Milford, MA, USA) and then centrifuged at 13,000× *g* for 1 min. The supernatant was discarded and the pellet resuspended in 1 mL of PBS. Samples were serially diluted in Luria–Bertani (LB) broth (Thermo Fisher Scientific Chemicals, Inc., Fair Lawn, NJ, USA) and plated on sorbitol MacConkey agar (SMAC; Thermo Fisher Scientific Chemicals, Inc.) and incubated at 42 °C overnight. *E. coli* colonies were selected for each sample from the SMAC plates and DNA extracted using a Qiagen DNeasy PowerFood Microbial Kit (Qiagen, Germantown, MD, USA) according to the manufacturer’s instructions. DNA concentration and quality measurements were taken with a Denovix DS-11 FX+ spectrophotometer.

### 2.3. Cold Sample Experiment

An experiment was conducted utilizing reagents that had been refrigerated overnight and then kept on ice during sample processing. Treatments were prepared in triplicate in sterile 7 oz Whirl-Pak^®^ filter bags containing 3 mL cold 2% sorbitol-mTSB. The treatments consisted of a media control, a positive control with no meat and 10^0^ cfu *E. coli*, a meat control with 1 g of uninoculated ground beef, and 1 g of ground beef inoculated with either 10^1^ or 10^0^ cfu g^−1^ *E. coli*. The treatments were kept on ice until they were homogenized with a stomacher. The samples were processed as described above for the small-scale experiment, except that the samples were kept on ice between steps and centrifugation occurred at 4 °C. Colonies were counted for each plate to confirm spiked concentrations, but no DNA extractions were performed.

### 2.4. Large-Scale Experiment

Three independent experiments were conducted with 5 treatments prepared in sterile 7 oz Whirl-Pak^®^ filter bags containing 30 mL cold 2% sorbitol-mTSB. Treatments included a media control, a positive control with no meat and 10^2^ cfu *E. coli*, a meat control with 10 g of uninoculated ground beef, and 2 treatments each with 10 g of ground beef and either 10^1^ or 10^0^ cfu g^−1^ *E. coli*. The treatments were then processed as described above for the small-scale experiment, except that samples were kept on ice between processing steps and centrifugation occurred at 4 °C.

### 2.5. Sequencing

Sequencing libraries were prepared with the Rapid Barcoding Kit (SQK-RBK114, Oxford Nanopore Technologies [ONT], Oxford, United Kingdom) using the DNA extracted from the *E. coli* colonies according to the manufacturer’s instructions. Sequencing was performed on a GridION device (ONT) with R10.4.1 flow cells (ONT) for 20 h. The sequencing data were basecalled in real time with MinKNOW software (version 23.11.7, ONT) using fast basecalling and a minimum read length filter of 1 kb.

### 2.6. Bioinformatics

The Galaxy platform was used to analyze the fastq files [32]. First, sequencing adapters were removed with Porechop (version 0.2.4). The resulting data was run through Fastp (version 0.23.4) using a Phred quality cutoff of 8 and minimum length of 1 kb. After quality control, the data was assessed with Nanoplot (version 1.42.0). The sequences were queried against a BLAST database consisting of the genes *fliC*, *eae*, *rrsC*, *stx1A*, *stx1B*, *stx2A*, *stx2B*, *wzx*, *wzy*, *espK*, and *espV* using NCBI BLAST + blastn (version 2.14.1). The number of times each gene was detected was summarized with the tool Count Occurrences of Each Record (version 1.0.3).

### 2.7. Statistics

The mean and standard error were calculated for the triplicate selective agar plate counts from the experiments. Statistical differences (*p* < 0.05) between the amounts of *E. coli* inoculated and recovered were determined with a one sample *t*-test. The mean and standard deviation were calculated for the number of times each gene of interest was detected in sequencing data.

## 3. Results

### 3.1. Small-Scale Experiment

*Escherichia coli* O157:H7 was inoculated into ground beef at concentrations ranging from 10^0^ to 10^3^ cfu g^−1^. No *E. coli* was isolated from the media or meat control treatments. However, the amount of *E. coli* recovered after plating was significantly higher (*p* < 0.05) than the inoculated amount, with most samples having a 1 log increase in *E. coli* concentration (Table 1). Colonies were selected from the SMAC agar plates for each concentration, and the DNA extracted and sequenced. An average of 0.52 Gb of sequencing data was produced from each colony. The metrics for the sequencing run are presented in Supplementary Table S1. All genes of interest, *fliC*, *eae*, *rrsC*, *stx1A*, *stx1B*, *stx2A*, *stx2B*, *wzx*, *wzy*, *espK*, and *espV* were detected an average of 168 times each in all samples (Table 2).

### 3.2. Cold Sample Experiment

The increase in *E. coli* concentration observed during the small-scale experiment prompted a study to determine if keeping samples cold during processing would prevent growth. Cold mTSB was used for dilutions, samples were placed on ice between processing steps, and centrifugation was conducted at 4 °C. Keeping samples cold prevented growth during sample preparation and there was no significant difference (*p* < 0.05) between the amount of *E. coli* inoculated into the sample and the amount recovered (Table 3).

### 3.3. Large-Scale Experiment

*Escherichia coli* O157:H7 was inoculated at 10^0^ and 10^1^ cfu g^−1^ into 10 g of ground beef, which was 10 times the amount used in the small-scale experiment. No *E. coli* was isolated from the media or meat control treatments. There was no significant difference (*p* < 0.05) in the concentration of *E. coli* inoculated into the treatments as compared to the amount observed on plate counts (Table 4). An average of 0.74 Gb of sequencing data was generated from each colony. Supplementary Table S2 shows the metrics of the sequencing run. All virulence genes of interest were detected an average of 206 times from each colony tested (Table 5).

## 4. Discussion

This study investigated whether the integration of serial plating with long-read sequencing could be used for foodborne pathogen testing. Host bovine DNA was eliminated from sequencing by extracting DNA from single colonies isolated on selective agar. Filtering the sample post-homogenization removed the larger eukaryotic bovine cells, and without the presence of lipid-rich ground beef, the remaining prokaryotic cells formed a tight pellet during centrifugation, allowing all supernatant to be removed prior to subsequent serial dilution and plating. Colonies exhibiting typical *E. coli* morphology on SMAC agar were selected for sequencing analysis. Contrary to concerns that a single bacterial colony might not yield enough DNA for sequencing, an average of 33 ng µL^−1^ of DNA was extracted, generating an average of 201,995 sequencing reads.

There is a need not only to identify pathogens in food samples but also to quantify their concentration to inform risk assessments and guide mitigation strategies. During the initial small-scale experiment, it was observed that the *E. coli* concentration on the serial dilution plates overestimated the inoculated amount. It was suspected that replication occurred during sample processing, as it was conducted at room temperature (~21 °C). Therefore, in subsequent experiments, reagents and samples were kept on ice, and centrifugation was performed at 4 °C. This modification prevented growth and ensured that the concentration of *E. coli* in the inoculum was accurately recovered. Current culture-based methods involve an enrichment step to ensure very low concentrations of pathogenic *E. coli* are detected [12], but this prevents absolute quantification of bacteria in the original sample. This study eliminated the enrichment step, and concentrations of *E. coli* as low as 1 cfu g^−1^ could be detected on selective agar, which is important because STEC are considered zero tolerance organisms in ground beef by FSIS [33]. Although enumeration of pathogens is not necessary to trigger a recall, it would improve risk management in the food industry. Quantification is important for assessing the effectiveness of sanitization procedures and evaluating quality assurance measures [34,35]. It can also be used to identify practices or products with high bacterial loads and trends in pathogen concentrations [36,37]. This information can help determine when to apply interventions or modify procedures to reduce bacterial loads.

The selective plating used in this study to isolate *E. coli* also overcomes one of the disadvantages of sequencing for foodborne pathogen testing, which is the lack of discrimination between live and dead cells. Interventions are often used to kill microorganisms in food, and the detection of DNA from these dead bacteria would result in a false positive and a potentially unnecessary recall [38]. Testing methods need to identify only live pathogens that may cause disease if consumed. Propidium monoazide treatment of bacteria prior to DNA extraction has been shown to prevent sequencing of dead bacteria, but it is still not quantitative [39]. The inclusion of serial plating in this study ensured that only live bacteria were selected for sequencing while also being quantitative.

Selecting only one morphologically typical colony for sequencing may be disadvantageous if multiple serotypes with indistinguishable morphologies are present in a sample. Current culture-based methods also require only one colony with typical morphology be selected for presumptive identification, isolation, and confirmation [12]. Reports of meat contamination with multiple serotypes are not common. A microbiological baseline study on veal found 12% of post-hide removal samples and 11% of post-chill samples tested positive for more than one non-STEC serotype [40]. Another study reported that 1% of *E. coli* outbreaks in the United States between 2010 and 2017 were attributed to both *E. coli* O157 and another non-O157 serotype [41]. However, the selection of only one isolate for testing may have missed other incidences involving multiple pathogenic serotypes. An advantage of sequencing over culture-based methods is that more colonies can be screened with less labor. Multiple colonies, either from the same sample or different samples, can be sequenced on the same flow cell. Each colony receives a unique barcode during library preparation, allowing sequencing data to be attributed back to a particular isolate. This is important because the pathogenic potential of *E. coli* depends on the specific virulence genes it carries [18]. Identification of *stx*, *eae*, and relevant serogroup genes within the same isolate is necessary to determine if the *E. coli* is STEC.

There were additional limitations to this study beyond the potential issue of detecting multiple serotypes, as discussed above. First, the samples from the cold experiment were not sequenced due to budget restraints. We did not anticipate sequencing data from *E. coli* processed under cold conditions to differ from those processed at room temperature because the genome, not transcripts, was being sequenced. However, this does represent a missed opportunity for additional data. This study also focused solely on *E. coli* in ground beef. Other pathogens or food matrices may perform differently due to variability in bacterial binding to a matrix, presence of background microflora, or bacteria that are viable but not culturable. Future experiments should also include mixtures of live and dead bacteria to further demonstrate the method’s ability to detect only live bacteria. Additionally, long-read sequencing technology changes rapidly, and revalidation of the method would be necessary when new library preparation kits, flow cell chemistries, or software are introduced.

## 5. Conclusions

This study established a method for foodborne pathogen testing that integrates serial dilution and plating with long-read sequencing. This approach effectively resolves critical challenges encountered in previous methods, such as host DNA interference, by enabling the isolation and sequencing of pathogen DNA from single colonies. Additionally, it allows for accurate quantification of live pathogens at low concentrations (1 cfu g^−1^), eliminating the need for enrichment steps that prevent true enumeration, and provides a clear distinction between viable and non-viable cells, thereby reducing false positives. Multiple colonies can also be sequenced in a single run, and the whole process takes three days. This method could be a powerful tool for improved food safety risk assessment and guiding more targeted intervention strategies within the food industry.

## Figures and Tables

**Table 1:** The concentration of *Escherichia coli* (cfu) inoculated into the treatments and the mean ± standard error *E. coli* (cfu) recovered on agar plates after processing in the small-scale experiments.

*E. coli* Inoculated (cfu)	Mean ± SE *E. coli* Recovered (cfu)
10^3^	13,167.0 ± 693.6
10^2^	864.3 ± 81.8
10^1^	181.3 ± 19.3
10^0^	22.0 ± 1.7

**Table 2:** The mean ± standard deviation of the number of times each gene of interest was detected in sequencing data from the small-scale experiment.

*Escherichia coli* (cfu g^−1^)	*fliC*	*eae*	*espK*	*espV*	*rrsC*	*stx1A*	*stx1B*	*stx2A*	*stx2B*	*wzx*	*wzy*
10^3^ *	90.7 ± 13.8	142.0 ± 22.5	100.5 ± 0.7	62.5 ± 3.5	624.3 ± 59.8	96.7 ± 11.4	82.7 ± 9.5	101.7 ± 9.3	93.3 ± 11.0	46.3 ± 10.4	34.7 ± 12.1
10^3^	94.7 ± 6.0	121.3 ± 4.2	157.5 ± 10.6	64.0 ± 6.7	708.0 ± 25.4	79.0 ± 11.1	71.3 ± 8.4	72.7 ± 19.1	96.7 ± 9.9	59.0 ± 7.0	37.0 ± 8.9
10^2^	65.3 ± 7.5	89.7 ± 2.9	115.5 ± 2.1	50.0 ± 7.1	671.0 ± 81.6	50.7 ± 6.8	45.0 ± 5.6	105.3 ± 2.1	68.0 ± 18.7	31.0 ± 3.6	29.7 ± 7.6
10^1^	171.3 ± 28.5	147.7 ± 42.8	70.0 ± 28.3	38.0 ± 4.2	1227.0 ± 45.0	107.3 ± 13.7	101.7 ± 15.9	207.0 ± 28.6	170.7 ± 24.8	79.7 ± 15.7	75.0 ± 15.5
10^0^	108.0 ± 10.5	290.3 ± 40.7	58.0 ± 1.4	54.0 ± 15.6	917.0 ± 64.9	150.0 ± 17.0	140.7 ± 12.1	134.0 ± 15.7	115.0 ± 6.1	112.7 ± 15.0	104.0 ± 16.4

* Positive control with no ground beef.

**Table 3:** The mean ± standard error of *Escherichia coli* recovered on agar plates after processing in the cold experiment.

*E. coli* Inoculated (cfu)	Mean ± SE *E. coli* Recovered (cfu)
10^3^	1433.0 ± 120.2
10^2^	133.3 ± 8.8
10^1^	20.3 ± 2.4
10^0^	4.7 ± 1.2

**Table 4:** The mean ± standard error of *Escherichia coli* recovered on agar plates after processing in the large-scale experiment.

*E. coli* Inoculated (cfu)	Mean ± SE *E. coli* Recovered (cfu)
10^1^	31.0 ± 5.2
10^0^	3.3 ± 1.8

**Table 5:** The mean ± standard deviation of the number of times each gene of interest was detected in sequencing data from the large-scale experiment.

*Escherichia coli* (cfu g^−1^)	*fliC*	*eae*	*espK*	*espV*	*rrsC*	*stx1A*	*stx1B*	*stx2A*	*stx2B*	*wzx*	*wzy*
10^2^ *	67.7 ± 12.9	79.7 ± 13.6	69.5 ± 12.0	67.0 ± 8.5	711.0 ± 70.2	47.7 ± 9.9	59.7 ± 8.7	83.0 ± 6.2	81.0 ± 8.2	25.0 ± 8.7	28.7 ± 7.6
10^1^	118.0 ± 13.1	131.0 ± 26.9	106.0 ± 2.3	142.5 ± 17.7	1191.0 ± 88.2	104.7 ± 15.5	116.3 ± 21.8	198.3 ± 17.6	210.7 ± 28.2	76.0 ± 9.0	68.3 ± 6.7
10^0^	161.3 ± 17.2	240.7 ± 13.3	158.0 ± 7.1	76.0 ± 18.4	1444.0 ± 65.7	146.0 ± 18.4	116.3 ± 21.1	144.0 ± 21.4	135.3 ± 21.6	100.7 ± 10.4	86.3 ± 17.2

* Positive control with no ground beef.

## Data Availability

The data presented in this study are openly available in USDA at https://doi.org/10.15482/USDA.ADC/31258642 [42].

## Supplementary Materials

The following supporting information can be downloaded at: https://www.mdpi.com/article/10.3390/pathogens15060573/s1, Table S1: Metrics of the sequencing data from the small-scale experiment; Table S2: Metrics of the sequencing data from the large-scale experiment.

## Abbreviations

The following abbreviations are used in this manuscript:

STEC	Shiga-toxin-producing *Escherichia coli*
U.S.	United States
USDA FSIS	U.S. Department of Agriculture Food Safety and Inspection Service
MALDI-TOF	Matrix-assisted laser desorption/ionization time-of-flight
mTSB	Tryptic soy broth modified with novobiocin
OD	Optical density
PBS	Phosphate-buffered saline
LB	Luria–Bertani broth
SMAC	Sorbitol MacConkey agar
CFU	Colony-forming unit
