# Supplementary material for: Enumeration and Genomic Confirmation of Viable Shiga-Toxin-Producing Escherichia coli from Ground Beef by Integrating Serial Plating with Long-Read Sequencing
Source: Pathogens. 2026 May 27;15(6):573. doi: 10.3390/pathogens15060573 (PMC13306003; doi:10.3390/pathogens15060573)
Supplement: Supplementary file 1 [file pathogens-15-00573-s001.zip › pathogens-4187109-supplementary.pdf]

Supplemental Data

Table S1. Metrics of the sequencing data from the small-scale experiment.

Escherichia coli **(cfu g^{-1})**	**Read Length Mean**	**Read Length Minimum**	**Read Length Maximum**	**Read Length Standard Deviation**	**Mean Estimated Bases (Gb)**	**Mean Reads Generated**	**Mean PHRED Score**	**Mean Estimated N50**
10^3*	3381	1000	20,004	2387.5	0.45	133,497	10.5	4356
10^3	3108	1000	20,259	2221.1	0.42	135,544	10.5	3899
10^2	2672	1000	11,351	1564.3	0.35	134,371	10.5	3195
10^1	3311	1000	19,764	2441.6	0.69	209,041	10.5	4240
10^0	3600	1000	21,310	2601.9	0.67	188,515	10.6	4673

*Positive control with no ground beef

Table S2. Metrics of the sequencing data from the large-scale experiment.

Escherichia coli **(cfu g^{-1})**	**Read Length Mean**	**Read Length Minimum**	**Read Length Maximum**	**Read Length Standard Deviation**	**Mean Estimated Bases (Gb)**	**Mean Reads Generated**	**Mean PHRED Score**	**Mean Estimated N50**
10^2*	3200	1000	20,970	2144.8	0.49	152,989	10.3	4021
10^1	3394	1000	19,744	2316.2	0.83	245,847	10.3	4334
10^0	2699	1000	14,165	1670.0	0.90	332,554	10.4	3224

*Positive control with no ground beef
